# Assessment of chicken thigh meat quality of Ross 308 broiler of animal welfare certified farm

**DOI:** 10.5713/ab.22.0044

**Published:** 2022-06-30

**Authors:** Hee-Jin Kim, Dong-Jin Shin, Hye-Jin Kim, Jinwoo Cho, Ji-Seon Kwon, Dongwook Kim, Jong-Hyun Jung, Aera Jang

**Affiliations:** 1 Poultry Research Institute, National Institute of Animal Science, Rural Development Administration, Pyeongchang 25342, Korea; 2 Department of Applied Animal Science, College of Animal Life Science, Kangwon National University, Chuncheon 24341, Korea; 3 R&D Material Research, Shinsegae Food Co., Ltd, Seoul 04793, Korea; 4 Jung P&C Institute, Inc., Yongin 16950, Korea

**Keywords:** Chicken, Dipeptides, Meat Quality, Ross 308, Welfare

## Abstract

**Objective:**

This study aimed to evaluate the difference in the thigh meat quality of Ross 308 broiler from conventional and welfare farms.

**Methods:**

Thigh meat samples of Ross 308 broilers (age, 35 d; carcass weight, 1.1 kg) from conventional farm (RCF, n = 60) and animal welfare farms (RAWF, n = 60) were analyzed. Proximate composition, pH, color (lightness, redness, and yellowness), water-holding capacity (WHC), shear force, total aerobic bacteria (TAB), and volatile basic nitrogen (VBN) were measured and the levels of bioactive compounds such as dipeptides (anserine and carnosine), creatine, creatinine, and their anti-oxidation activity were determined.

**Results:**

The RCF and RAWF did not differ significantly in their proximate composition, WHC, color, and creatine and carnosine levels. The pH value was significantly lower in RAWF than in RCF on day 7. The shear force value was significantly higher in RAWF than in RCF throughout the storage duration. TAB in RCF on day 9 were significantly higher than those in RAWF. The VBN content of RAWF was significantly lower than that of RCF after 5 days of storage. Creatinine content was significantly higher in RAWF (3.50 mg/100 g) than in RCF (3.08 mg/100 g) on day 1. Along with higher carnosine and anserine contents of RAWF, it had significantly higher 2,2-diphenyl-1-picrylhydrazyl and 2,2-azinobis (3-ethyl-benzothiazoline-6-sulfonic acid) radical scavenging activities than those of RCF.

**Conclusion:**

These results imply that the animal welfare farming system beneficially affects the overall oxidative stability of Ross 308 thigh meat.

## INTRODUCTION

Chicken meat is a highly nutritional food source. It contains abundant high-quality protein, bioavailable iron, and essential fatty acids with low calorific values and high unsaturated fatty acid content. It also contains endogenous bioactive compounds such as creatine and dipeptides (anserine and carnosine), which are found commonly in meat [1]. Chicken meat consumption has been increasing with increasing population, per capita income, and changing lifestyle of health-conscious consumers [2]. Additionally, consumer demand has also diversified. In recent times, animal welfare has become a concern for consumers, policy makers, and scholars worldwide [3].

Animal husbandry practices require controlled air and litter quality, stocking density, lighting, and slaughtering to achieve animal welfare [4]. These practices can influence meat quality and consequently the economical aspect of meat production. For example, a decreased stocking density can relieve stress and aggression for chickens but increase mortality than indoor housing system [5,6]. Also, some reported that the meat from free-range chickens was juicer [6]. Significant variations in meat color and water holding capacity were observed during processing and transportation, depending on the type of bird, season, and the scale of retail activity [7,8].

There is limited evidence of animal welfare farming system affecting the meat quality and bioactive compounds of chicken meat. To our knowledge, there have been no studies on evaluating the bioactive compounds in chicken meat from Ross 308, one of the most popular breeds, while studies about Arbor acres [9] and Cobb species [10] have been reported. Therefore, this study was conducted to comparatively analyze the bioactive compounds and meat quality properties of Ross 308 thigh meat obtained from different farming system (conventional and animal welfare farm) during cold storage.

## MATERIALS AND METHODS

### Birds and meat sampling

Chicken broilers (Ross 308) which were reared by animal welfare farming and conventional farming (n = 60, respectively; carcasses weighing 1.1 kg) were randomly collected and slaughtered in a local commercial slaughterhouse (Charmfre Co., Buan, Korea). Then, the thigh meat was isolated and stored at 4°C for 9 days. Analyses were conducted on day 1, 3, 5, 7, and 9 of storage. The conditions for conventional farming and animal welfare farming [11] are presented on Table 1.

### Proximate analysis and pH

The proximate composition (moisture, crude protein, crude fat, and crude ash) of thigh meat was evaluated using the AOAC methods [12]. Approximately 10 g of meat was homogenized with 90 mL of distilled water (DW) for 60 s in a homogenizer (PolyTron PT-2500 E; Kinematica, Lucerne, Switzerland). pH value of the meat was determined using a digital pH meter (Orion 230A; Thermo Fisher Scientific, Inc., Waltham, MA, USA).

### Color

Color of broiler chicken thigh meat was assessed using a colorimeter (Chroma Meter CR-400; Minolta Co., Osaka, Japan) using the lightness (CIE L*), redness (CIE a*), and yellowness (CIE b*) parameters. The colorimeter was calibrated using the standard white plate (Y value, 93.60; x value, 0.3134; y value, 0.3194).

### Water-holding capacity

The water-holding capacity (WHC) was evaluated according to the method described by Kim et al [9]. Approximately 0.5 g sample was placed on a round plate in a Millipore tube (Millipore Ultrafree-MC; Millipore, Bedford, MA, USA) and heated in a water bath at 80°C for 20 min. It was cooled to 23°C and centrifuged (2,000×g) at 4°C for 10 min to measure the water loss.

### Shear force

Thigh meat of broiler was placed in a water bath in a polyethylene bag until the internal temperature of the meat reached 75°C. Then, after cooling for 20 min at 23°C, thigh meat samples were sheared in the orientation of the fibers of the muscle, in subsamples of about 1×2×1 cm. Shear force values were determined using the V-blade using Texture Analyzer TA 1 (LLOYD instruments, Berwyn, IL, USA). The Texture Analyzer settings were as follows: test speed, 50 mm/min; trigger force, 0.01 kgf; trigger speed, 50 mm/min; load cell, 500 N.

### Microbial content

Total aerobic bacteria (TAB) count was measured using 5 g meat homogenized with 45 mL distilled water by pummeling in a stomacher (Bag Mixer 400; Interscience, St. Nom, France) for 2 min. Microorganisms (aerobic count plate and *E. coli*/coliform) were determined using 3M Petrifilm (Bioser, Barcelona, Spain) after incubating at 37°C for 48 h using manufacturer’s protocol. Three replicate trials were conducted for each sample. Results were expressed as log colony-forming unit (CFU)/g.

### Total volatile basic nitrogen

Microdiffusion method was used to evaluate total volatile basic nitrogen (VBN) as described by Kim et al [13]. Each meat sample (10 g) was homogenized with 50 mL DW for 30 min using a magnetic stirrer. The solution was filtered using a filter paper (No. 1; Whatman, Clifton, NJ, USA). One milliliter of H_2_SO_4_ (0.01 N) was placed in the inner section of a Conway micro-diffusion cell (Sibata Ltd., Sitama, Japan). One milliliter of sample solution and 1 mL of saturated K_2_CO_3_ were placed on the outer part of the same cell and covered immediately. The Conway micro-diffusion cell was incubated at 30°C for 60 min, and it was then titrated against 0.01 N NaOH. The total VBN content was reported as mg/100 g of sample.

### 2-thiobarbituric acid reactive substances

The 2-thiobarbituric acid reactive substances (TBARS) was measured by using a modification of the procedure reported by Lee et al [14]. The DW (15 mL) with 7.2% tert-butyl-4-hydroxyanisole (50 μL) was added to the meat (5 g) and homogenized using a homogenizer (Polytron PT-2500E; Kinematica, Lucerne, Switzerland). One milliliter of homogenate solution was mixed with 2 mL of 20 mM thiobarbituric acid (in 15% trichloroacetic acid). The mixture was heated in water bath at 90°C for 15 min and then cooled for 10 min. The sample was then centrifuged at 2,000×g (4°C, 10 min), and absorbance of supernatant was measured at 531 nm using a spectrophotometer (Molecular Device, M2e, Sunnyvale, CA, USA). The amount of TBARS was expressed as mg of malondialdehyde (MDA) per kg meat.

### Creatine, creatinine, and dipeptides (anserine and carnosine)

The creatine, creatinine, and di-peptide (anserine and carnosine) content were determined using the method described by Mora et al [15]. Lyophilized thigh meat (2.5 g) was homogenized with 7.5 mL of 0.01 N HCl for 1 min. After homogenization, the sample was centrifuged for 30 min (3,000×g, 4°C). Then, the supernatant was filtered using a glass microfiber filter (Whatman GF/C, Maidstone, Kent, UK), and 250 μL of filtrate was combined with 750 μL of acetonitrile. The solution was left for 20 min and then centrifuged for 10 min at 10,000×g (4°C). The supernatant was filtered using a 0.22 μm membrane filter, and 20 μL of filtered sample was injected into an Atlantis HILIC silica column (150×4.6 mm, 3.0 μm; Waters, Milford, MA, USA) in the Agilent Infinity 1260 series HPLC (Agilent Technologies, Palo Alto, CA, USA) system. The creatinine was determined at 236 nm and creatine and dipeptide (anserine and carnosine) were assayed at 214 nm. Mobile phases consisted of solvent A (0.65 mM ammonium acetate in water [pH 5.5]/acetonitrile [25:75]) and solvent B (4.55 mM ammonium acetate in water [pH 5.5]/acetonitrile [70:30]). The solvent B was supplied at 1.4 mL/min with a linear gradient (0% to 100%) for 13 min. Standard reagent of creatine, creatinine, and dipeptide (anserine and carnosine) were purchased from Sigma-Aldrich (St. Louis, MO, USA) and all chemicals used were of analytical grade.

### Antioxidation activity

The 2,2 Diphenyl-1-picrylhydrazyl (DPPH) radical scavenging activity of meat was estimated according to a method described by Blois [16]. The sample (0.5 mL) was mixed with 0.5 mL of 0.2 mM DPPH solution. Subsequently, the mixture was left in the dark at 23°C for 30 min. The absorbance of the mixture was measured at 517 nm using a spectrophotometer (SpectraMax M2e; Molecular Devices, Sunnyvale, CA, USA). ABTS [2,2′-azinobis-(3-ethylbenzothiazoline-6-sulfonic acid)] radical scavenging activity was determined using the method described by Re et al [17]. ABTS working solution was obtained by mixing 2.45 mM of K_2_S_2_O_8_ solution and 7 mM ABTS^+^ solution and incubating the mixture for 16 h at 23°C in the dark. The mixture was diluted with DW to obtain an absorbance of 0.700 at 735 nm. Sample (50 μL) was allowed to react with 950 μL fresh ABTS^+^ solution. Then, the mixture was left for 30 min at 30°C in the dark. Absorbance was measured at 735 nm. Ferric reducing antioxidant power (FRAP) activity was assessed by modifying the method described by Benzie and Strain [18]. FRAP solution was prepared by mixing 300 mM acetate buffer, 20 mM FeCl_3_, and 10 mM TPTZ in 40 mM HCl in a ratio of 10:1:1 (v/v). Sample (25 μL) was mixed with 175 μL of FRAP solution at 37°C for 30 min. The absorbance was recorded at 590 nm. Oxygen radical absorbance capacity (ORAC) was determined using the method reported by Gillespie et al [19]. ORAC measured using a fluorescence detector with emission and excitation wavelengths of 485 and 520 nm, respectively, using a microplate reader every minute for 60 min at 37°C.

### Statistical analysis

All the analysis were done more than triplicate and data were analyzed by SAS (ver. 9.4; SAS Institute Inc., Cary, NC, USA) using one-way analysis of variance and the generalized linear model. Differences in mean values were analyzed using Tukey’s range tests (p<0.05). The data were expressed as mean value and standard error of mean.

## RESULTS AND DISCUSSION

### Proximate composition

No significant differences in moisture (74.51% to 76.02%), crude protein (18.37% to 18.74%), crude fat (6.25% to 6.45%), or crude ash (1.50% to 1.70%) composition was found between Ross 308 from conventional farms (RCF) and Ross 308 from animal welfare farms (RAWF) (Table 2). This finding is like the results of Kim et al [13] wherein the moisture level, crude protein, crude fat, and crude ash content of thigh meat obtained from Arbor Acers broilers experiencing different farming systems (welfare farm vs conventional farm) did not differ significantly. Husak et al [20] reported that moisture, protein, or fat contents of broiler thigh meat obtained from organic, free-range, and conventionally farmed chickens were similar, and ranged between 72.56% to 73.99%, 17.82% to 19.25%, and 5.92% to 7.23%, respectively. Moisture and fat composition analyses results of meat obtained for this study were not significantly different from organic, free-range, and conventionally farmed chickens. However, protein content of thigh meat from conventionally farmed chickens were significantly lower than that of those from organic and free-range farm chicken [20].

### Physicochemical properties

Physicochemical characteristics of chicken thigh meat of RCF and RAWF are shown in Table 2. It has been reported that chronic stressors influence muscle glycogen levels [14]. Higher muscles glycogen content during slaughter resulted in lower final pH than that in animals with less glycogen because glycogen is changed to lactic acid [14]. However, the pH values of thigh meat on days 1, 3, 5, and 9 did not differ significantly in the chicken from the different farming systems. Consistent with our results, Husak et al [20] found that the pH values of thigh meat from organic and conventional farm were not different on day 1. Goo et al [21] reported that pH of chicken meat was not affected stocking density (15.2, 20.2, 25.3, or 30.4 birds/m^2^). Tuell et al [22] investigated the effect of photoperiod length (L = light, D = Dark, 20 L:4 D, 18 L:6 D, 16 L:8 D, or 12 L:12 D) on chicken meat and reported that photoperiod had no impact on meat pH. In this study, this suggested that the farming system such as that level of stocking density and photoperiod did not affect pH of meat. The pH of RCF increased significantly, while the that of RAWF did not change statistically during storage. Silva and Glória [23] reported that the pH of thigh meat was not significantly different on days 1 and 10. Also, Hulankova et al [24] found that thigh and breast meat were not significantly different in pH over 14 days during refrigerated storage. In contrast, Kim et al [10] reported that the increase in pH of chicken meat during storage. These discrepancies in chicken pH values may be related to differences in initial microbial composition producing microbial metabolites such as amines, ammonia, and lactic acid during storage [14].

The WHC is important because water retention and loss can influence the weight and economic value of chicken meat products. The WHC of meat is defined as the property of maintaining moisture when the meat is exposed to an external physical exertion such as pressing, cutting, grinding, or heat treatment [14]. No significant difference was found in WHC of RCF and RAWF (WHC ranged from 56.10% to 60.98%). WHC of meat is affected by moisture content and pH value of the meat [25]. As shown in Tables 2 and 3, the moisture content and pH of the thigh meat did not differ between the two farms and resulted in no difference in WHC of RCF and RAWF. However, Lee et al [14] reported that pH and WHC of Cobb chicken leg meat from certified animal welfare farm was significantly lower than from conventional farm on day 1 and 3. The difference could be due to difference of breeds between Ross 308 and Cobb [26]. Further study needs to evaluate WHC characteristics of chicken thigh meat from animal welfare farms.

Meat buying decisions are affected more by the meat color than by other quality factors, as consumers conceive the changed color as an indicator of intactness and freshness [25]. The L*, a*, and b* (lightness, redness, and yellowness, respectively) of RCF and RAWF remained constant value and showed no significant difference for 9 days. Husak et al [20] reported that the L* values of thigh meat of chicken from free-range farm did not differ from that of thigh meat of conventionally farmed chicken. Castellini et al [27] found that the a* and b* values of Ross broiler thigh meat from organic and conventional farms were not different at 56 d of age. Fanatico et al [28] reported that the color of a particular broiler species (Cobb) remained the same regardless of the farming system (organic, free-rang, or conventional). It is well known that the chicken meat color is related with WHC and ultimate pH of meat [25]. We found that the WHC and pH values of thigh meat from RCF and RAWF showed no significant difference. This can be the reason why the similar color values of thigh meat from RCF and RAWF.

Shear force is indicative of the state of myofibrillar protein and connective tissue and contributes to meat preference and cooked meat tenderness [29]. The shear force of thigh meat of the chicken from welfare farm was significantly higher than that of meat from conventional farm during whole storage days, indicating the effect of nature of farming system on the shear force. Similarly, Husak et al [20] reported that shear force of thigh meat from organic farm was higher than that of thigh meat from conventional farm. Moreover, Sun et al [30] reported that the shear force of free-range broiler breast meat was significantly higher than that of indoor broiler breast meat due to difference in the locomotory activity. Castellini et al [27] suggested that free-range farming systems affect the shear force, owing to the greater physical movement of broilers in free-ranging farming systems. The initial shear force (day 1) for RCF and RAWF thigh meat was 22.43 to 25.17 N that decreased significantly during storage (Table 3). The shear force decreases with increase in storage period, as muscle protein gets decomposed by endogenous and microbial enzymes [31].

### Microorganisms, TBARS, VBN value

The counts of TAB and coliform in meat are useful indicators of microbial contamination and thus of hygiene during processing and storage [32]. Chicken meat with a high number of bacteria results in poor processed products with a shorter shelf-life. Microorganism counts, TBARS, and VBN value of chicken thigh meat of RCF and RAWF are shown in Table 4. The TAB of RCF and RAWF increased significantly during storage from 2.48–2.61 log CFU/g on day 1 to 6.07–6.68 log CFU/g by day 9. The TAB count for RCF (6.68 log CFU/g) was higher than that for RAWF on day 9 (6.07 log CFU/g). TAB in all sample during storage was within the limit of 6.7 log CFU/g, which was under the regal guideline of TAB level by the Ministry of Food and Drug Safety of the Republic of Korea [33]. It confined the TAB level of retailed fresh meat (beef, pork, and chicken) from 5×10^6^ CFU/g (6.70 log CFU/g) to 1×10^7^ CFU/g (7 log CFU/g). The TAB value of thigh meat of RCF stored for 9 days was close to the permissible limit at 6.68 log CFU/g. There was no significant difference on both TAB and coliforms of thigh meat between RCF and RAWF broilers during storage. da Silva et al [34] reported that thermotolerant coliforms and mesophilic bacteria of broilers (Cobb and Ross strain) from free-range and industrial farms showed no difference on day 1.

TBARS of thigh meat from both RAWF and RCF broilers increased significantly during storage and reached up to 0.41 to 0.43 mg MDA/kg. Many studies reported that chicken meat becomes rancid when TBARS value exceeds 0.6 to 2.0 mg MDA/kg [25], since the value considered as the degree of lipid oxidation. We found that the TBARS values of thigh meat from RCF and RAWF were not significantly different and remained below 0.45 mg MDA/kg. However, several studies showed that animal welfare farming system reduced lipid oxidation of chicken meat of Arbor Acres [9] and Cobb [10] during cold storage.

The VBN value, indicating the production of protein-derived basic compounds, denotes the freshness of meat [25]. VBN value of chicken thigh meat increased with increase in storage duration (Table 4). The VBN value of chicken meat is known to be increased mainly by proteolysis of microorganisms [25,35]. The increase in VBN was due to increased TAB count during storage, as shown in this study. The VBN values of RAWF were significantly lower than those of RCF during only storage day 5 to 9. This result indicated that animal welfare certified farming system did not significantly affect the freshness of chicken thigh meat compared to conventional farming system between day 1 to 3. Although the VBN value of RAWF from day 5 to 9 was significantly lower than that of RCF, the VBN values of day 7 and 9 were 23.09 to 24.69 mg/100 g sample and 25.09 to 26.16 mg/100 g sample, respectively, exceeding the MFDS mandated VBN upper limit value (20 mg/100 g) for freshness of meat [36]. Thigh meat from both RCF and RAWF were considered decomposed already after day 7. Relatively lower VBN values of chicken meat from animal welfare farming than of those from conventional farming have been reported previously in breast meat of Arbor Acers [9,13] and Cobb [10] breeds. Kim et al [13] reported prolonged (>2 d) freshness of animal welfare farmed meat with respect to the VBN value.

### Creatine, creatinine, and di-peptide (anserine and carnosine)

Creatine, creatinine, anserine, and carnosine are bioactive compounds that cannot be supplied by plant-origin foods [35]. Along with its well-known functional effects on improvement of muscle tissue to enhance athletic performance, supplementation of creatine prevents neurodegenerative diseases [37]. Creatinine is the product of spontaneous conversion of creatine at a constant rate [38]. In this study, the contents of creatine, creatinine, and dipeptide of thigh meat of RCF and RAWF are shown in Table 5. The contents of creatine and creatinine in thigh meat of RAWF were 323.70 to 332.39 mg/100 g and 3.50 to 4.51 mg/100 g, respectively. No difference was found in creatine levels in broilers experiencing different farming conditions. However, creatinine of RAWF was significantly higher than that of RCF on day 1. Similar results were reported for thigh meats of Cobb broiler from animal welfare farm and conventional farm [10].

Carnosine and anserine consist of β-alanine and L-histidine. Anserine is an N-methylated derivative of carnosine. Both compounds have antioxidative, antiaging, and pH buffering properties [39]. The carnosine contents of RAWF were higher compared to that of RCF, especially on day 3. Anserine, was also significantly higher in RAWF than in RCF on day 7 and 9. The higher carnosine and anserine contents of RAWF could be owing to the allowance of more activity to birds by the lower stocking density and perch support on animal welfare farming. It was referred that muscle activity is closely related to the dipeptide concentration. The dipeptide concentration in vigorously contracting muscles was higher than the concentration in denervated muscles [40]. While the reported anserine content in meat produced in animal welfare farm varied temporally. Kim et al [13] observed relatively higher anserine contents in Cobb broiler meat from animal welfare farming. Anserine content of Abor Acre broiler meat from animal welfare farm was higher than that of meat from the conventional farm during cold storage (60 to 110 mg/100 g vs 58 to 81 mg/100 g) [9,17]. Anserine content of Cobb broilers from animal welfare farms was also higher than broilers of the conventional farms, although no significant difference could be found during storage [10]. However, Lee et al [41] reported significantly lower anserine content in pork from welfare farm. The anserine content in meat is affected by muscle type, species, sex, and age of the animal [13].

### Antioxidant activities

DPPH and ABTS radical scavenging activity, FRAP, and ORAC of thigh meat of RCF and RAWF is shown in Table 6. The DPPH radical scavenging activities of RAWF were higher than those of RCF during storage days, especially on day 1 and 9. Similarly, RAWF had significantly higher ABTS radical scavenging activities than RCF during storage except on day 9. This difference in antioxidant activity in the chicken meat was because of the difference in the contents of anti-oxidative compounds such as carnosine and anserine [42]. As the RAWF had higher concentration of these compounds, its DPPH and ABTS radical scavenging activities were also higher. This result is in accordance with the findings of Kim et al [13]. Kim and Jang [43] reported that the antioxidant activities of beef were also positively correlated with carnosine and anserine contents. In animal welfare certified farms, broiler diet is supplemented with plant source antioxidative compounds such as phenols and flavonoids. This possibly helps in enhancing the antioxidant activity of thigh meat of RAWF. It is well known that administration of the plant-derived phenolic compounds can improve antioxidative status of chicken [44]. Meanwhile, no significant difference of FRAP and ORAC activity between RAWF and RCF was found throughout the storage. Kim et al [13] reported higher FRAP activity in Cobb broiler thigh from animal welfare farm on day 1. However, ORAC activity of conventionally and animal welfare farmed broilers showed no difference on days 1, 5, 7, and 9 [13]. Further studies are needed to understand the discrepancy of antioxidant activities among different broiler species.

## IMPLICATIONS

The thigh meats of Ross 308 broilers from animal welfare farm had a higher shear force and lower microbial count than those from conventional farm. It was shown that animal welfare farming beneficially affected the overall oxidative stability of the chicken meat. However, some discrepancy was found in the parameters for oxidative stability compared to previous studies, maybe because of the difference in the breeds of broilers. Therefore, further experiments comparing the oxidative stability of different breeds of broiler would be needed to understand the influence of animal welfare farming on meat quality.

## Figures and Tables

**Table 1:** Difference in conditions between the conventional farm and animal welfare farm

Items	Conventional farm	Animal welfare farm
Stocking density	22 to 26 bird, 33 to 39 kg/m^2^	<20 bird, 30 kg/m^2^
Perch and pecking materials	Not provided	provided
Ammonia level	Not controlled	<25 ppm
CO_2_ level	Not controlled	<5,000 ppm
Photoperiod	No standard condition	<16 h light/d, >4 h dark/d
Light density	No standard condition	>20 lux
Diet	Conventional broiler diet	Protein derived from mammals or birds was not included^1)^

1) The crude protein level and apparent metabolizable energy of starter, grower, and finisher diet were equalized in both the feeds for conventional farm and animal welfare farm.

**Table 2:** Proximate composition of thigh meat of Ross 308 broilers from conventional and animal welfare farming system during cold storage

Items (%)	Treatment	Storage days					SEM

		1	3	5	7	9	
Moisture	RCF	74.63	74.51	74.80	75.20	74.87	0.594
	RAWF	74.97	74.99	75.01	75.88	76.02	0.444
	SEM	0.480	0.572	0.670	0.230	0.561	
Crude protein	RCF	18.49	18.51	18.45	18.46	18.66	0.421
	RAWF	18.37	18.74	18.56	18.74	18.69	0.354
	SEM	0.254	0.391	0.345	0.448	0.468	
Crude fat	RCF	6.37	6.25	6.07	6.29	6.40	0.404
	RAWF	6.31	6.31	6.45	6.70	6.27	0.291
	SEM	0.361	0.415	0.267	0.382	0.317	
Crude ash	RCF	1.55	1.67	1.59	1.61	1.61	0.089
	RAWF	1.50	1.70	1.58	1.63	1.56	0.084
	SEM	0.082	0.083	0.105	0.075	0.085	

SEM, standard error of means; RCF, Ross 308 from conventional farm; RAWF, Ross 308 from animal welfare farm.

**Table 3:** Meat pH, water holding capacity (WHC), instrumental color, and shear force of thigh meat of Ross 308 broilers from conventional and animal welfare farming system during cold storage

Items		Treatment	Storage days					SEM

			1	3	5	7	9	
pH		RCF	6.50^b^	6.57^ab^	6.64^ab^	6.67^Aab^	6.74^a^	0.051
		RAWF	6.51	6.54	6.56	6.59^B^	6.67	0.049
		SEM	0.060	0.078	0.029	0.024	0.038	
WHC (%)		RCF	58.70	56.75	59.19	58.64	60.98	1.881
		RAWF	57.00	56.31	60.01	56.10	58.73	1.632
		SEM	2.300	2.144	1.366	1.378	1.362	
Color	L*	RCF	53.80	53.64	53.45	54.48	53.59	0.909
		RAWF	52.68	53.80	52.87	54.94	53.23	0.944
		SEM	0.809	1.059	0.815	1.024	0.899	
	a*	RCF	7.68	7.20	6.94	6.90	6.38	0.505
		RAWF	9.28	8.06	8.27	8.26	8.07	0.527
		SEM	0.651	0.422	0.427	0.468	0.571	
	b*	RCF	7.69^c^	8.37^bc^	9.02^bc^	9.75^b^	11.67^a^	0.403
		RAWF	7.02^c^	7.97^bc^	8.94^b^	9.25^ab^	10.62^a^	0.355
		SEM	0.361	0.425	0.399	0.318	0.386	
Shear force (N)		RCF	22.43^Ba^	19.76^Bb^	18.27^Bb^	15.19^Bc^	14.17^Bc^	0.476
		RAWF	25.17^Aa^	22.69^Ab^	20.97^Abc^	19.71^Ac^	16.60^Ad^	0.557
		SEM	0.583	0.492	0.496	0.381	0.608	

SEM, standard error of means; RCF, Ross 308 from conventional farm; RAWF, Ross 308 from animal welfare farm.

A,B Means within the same column with different letters are significantly different (p<0.05).

a–d Means within the same row with different letters are significantly different (p<0.05).

**Table 4:** Microorganisms, 2-thiobarbituric acid reactive substances (TBARS), and volatile basic nitrogen (VBN) value of thigh meat of Ross 308 broilers from conventional and animal welfare farming system during cold storage

Items		Treatment	Storage days					SEM

			1	3	5	7	9	
Micro-organisms (log CFU/g)	Total aerobic bacteria	RCF	2.61^d^	2.64^d^	3.95^c^	5.01^b^	6.68^Aa^	0.089
		RAWF	2.48^d^	2.46^d^	3.81^c^	4.90^b^	6.07^Ba^	0.100
		SEM	0.111	0.103	0.053	0.079	0.112	
	Coliforms	RCF	ND^b^	ND^b^	ND^b^	1.31^a^	1.43^a^	0.156
		RAWF	ND^b^	ND^b^	ND^b^	1.27^a^	1.36^a^	0.148
		SEM	ND	ND	ND	0.140	0.164	
TBARS (mg MDA/kg)		RCF	0.18^d^	0.23^c^	0.31^b^	0.34^b^	0.43^a^	0.008
		RAWF	0.17^d^	0.23^c^	0.31^b^	0.30^b^	0.41^a^	0.008
		SEM	0.008	0.007	0.011	0.007	0.007	
VBN (mg/100 g)		RCF	4.87^e^	7.58^d^	14.30^Ac^	25.09^Ab^	26.16^Aa^	0.182
		RAWF	4.58^e^	7.14^d^	13.18^Bc^	23.09^Bb^	24.69^Ba^	0.237
		SEM	0.169	0.243	0.253	0.167	0.210	

SEM, standard error of means; RCF, Ross 308 from conventional farm; RAWF, Ross 308 from animal welfare farm; CFU, colony-forming unit; ND, not detected; TBARS, 2-thiobarbituric acid reactive substances; VBN, volatile basic nitrogen.

A,B Means within the same column with different letters are significantly different (p<0.05).

a–e Means within the same row with different letters are significantly different (p<0.05).

**Table 5:** Creatine, creatinine, carnosine, and anserine contents of thigh meat of Ross 308 broilers from conventional and animal welfare farming system during cold storage

Items (mg/100 g)	Treatment	Storage days					SEM

		1	3	5	7	9	
Creatine	RCF	334.28	337.23	312.43	313.49	307.28	9.511
	RAWF	332.39	325.13	323.70	326.81	328.32	13.476
	SEM	12.123	13.695	10.740	11.215	10.224	
Creatinine	RCF	3.08^Bb^	4.10^a^	4.29^a^	4.76^a^	4.43^a^	0.228
	RAWF	3.50^Ab^	4.38^a^	4.24^a^	4.42^a^	4.51^a^	0.136
	SEM	0.115	0.121	0.098	0.322	0.186	
Carnosine	RCF	40.12	43.52^B^	49.01	45.32	44.60	5.727
	RAWF	48.06	62.46^A^	63.79	54.24	55.36	4.902
	SEM	2.940	4.520	7.668	3.509	6.472	
Anserine	RCF	127.56	115.98	121.66	110.36^B^	107.16^B^	5.776
	RAWF	129.45	125.90	127.99	125.29^A^	118.01^A^	7.125
	SEM	8.702	7.715	6.980	3.930	3.305	

SEM, standard error of means; RCF, Ross 308 from conventional farm; RAWF, Ross 308 from animal welfare farm.

A,B Means within the same column with different letters are significantly different (p<0.05).

a,b Means within the same row with different letters are significantly different (p<0.05).

**Table 6:** Antioxidation activity of thigh meat of Ross 308 broilers from conventional and animal welfare farming system during cold storage

Items (μmol TE/g)	Treatment	Storage days					SEM

		1	3	5	7	9	
DPPH	RCF	11.81^Ba^	10.32^ab^	9.64^b^	9.28^b^	6.65^Bc^	0.410
	RAWF	14.32^Aa^	11.00^b^	10.63^bc^	8.05^c^	9.04^Abc^	0.674
	SEM	0.357	0.691	0.566	0.526	0.593	
ABTS	RCF	121.54^Bab^	121.85^Ba^	117.08^Bbc^	116.92^Bbc^	117.54^abc^	1.060
	RAWF	126.46^Aa^	125.08^Aab^	122.62^Aabc^	120.77^Abc^	119.38^c^	1.159
	SEM	1.343	0.644	1.462	1.176	0.658	
FRAP	RCF	11.79^a^	10.93^ab^	9.98^ab^	8.99^b^	8.63^b^	0.586
	RAWF	11.87^a^	10.25^ab^	8.99^b^	8.31^b^	8.74^b^	0.612
	SEM	0.636	0.949	0.385	0.457	0.365	
ORAC	RCF	242.37	228.14	209.97	211.84	194.81	12.673
	RAWF	259.70^a^	240.25^ab^	221.89^bc^	207.41^bc^	195.84^c^	8.256
	SEM	6.185	10.885	6.721	16.983	9.035	

SEM, standard error of means; RCF, Ross 308 from conventional farm; RAWF, Ross 308 from animal welfare farm; DPPH, 2,2 Diphenyl-1-picrylhydrazyl; ABTS, 2,2′-azinobis-(3-ethylbenzothiazoline-6-sulfonic acid; FRAP, ferric reducing antioxidant power; ORAC, oxygen radical absorbance capacity.

A,B Means within the same column with different letters are significantly different (p<0.05).

a–c Means within the same row with different letters are significantly different (p<0.05).
